# Omics datasets can bridge the gap between tumor biology and patient care

**DOI:** 10.1371/journal.pbio.3003279

**Published:** 2025-07-28

**Authors:** Ji-Ting Huang, Lei-Jie Dai, Ding Ma, Zhi-Ming Shao

**Affiliations:** 1 Department of Breast Surgery, Key Laboratory of Breast Cancer in Shanghai, Fudan University Shanghai Cancer Center, Shanghai, China; 2 Department of Oncology, Shanghai Medical College, Fudan University, Shanghai, China; Princeton University, UNITED STATES OF AMERICA

## Abstract

Large-scale omics datasets from tumor samples are becoming ever easier to generate and analyze. This Perspective looks at how, as technology continues to push forwards, multiomics data be leveraged to help patients in the clinic.

## Teaser

Large-scale omics datasets from tumor samples are becoming ever easier to generate and analyze. As technology continues to push forward, how can this data be leveraged to help patients in the clinic?

The rapid advancement of omics technologies—genomics, proteomics and beyond—has illuminated the molecular complexity of tumors, driving precision oncology forward. Despite these breakthroughs, knowing how to translate large omics datasets into clinically relevant applications remains a major impediment. Three key approaches have emerged: leveraging large-scale data for identifying therapeutic targets; refining molecular subtyping to guide tailored treatments; and integrating predictive modeling to optimize clinical decisions. Together, these approaches provide a potential solution to the challenge of applying omics discoveries to personalized interventions, bridging the gap between tumor biology and patient care.

The integration of large-scale omics data has already enabled the discovery of highly selective therapeutic targets in oncology. A notable example is Claudin 18.2 (CLDN18.2), a protein involved in tight junctions. In a seminal 2008 study, CLDN18.2 was identified through human expressed sequence tag files from the NCBI database, revealing its aberrant activation in a wide range of epithelial tumors, including gastric, pancreatic and esophageal cancers [1]. Its restricted expression in normal tissues and consistent presence in tumors made it a promising candidate for antibody-based therapy. This discovery led to the development of zolbetuximab, a monoclonal antibody specifically targeting CLDN18.2, which is now recommended as the first-line treatment for gastric cancer with high CLDN18.2 expression [2]. Additional therapeutic strategies targeting CLDN18.2, such as bispecific antibodies, antibody–drug conjugates and chimeric antigen receptor T cell therapies, are currently under investigation, illustrating how omics-guided target discovery can drive rational drug development and result in novel treatment options. Although omics-guided target discovery holds great promise, a major challenge remains in tumor heterogeneity, which encompasses both intratumoral cellular diversity and the dynamic changes that occur during tumor progression. Fortunately, advances in spatial technologies and liquid biopsy approaches, such as circulating tumor DNA (ctDNA) profiling, offer promising avenues to address the issue of heterogeneity.

The integration of omics data for molecular subtyping, with the aim of unraveling tumor heterogeneity, has been used to identify key molecular drivers for precision therapy. Early studies, such as those into the PAM50 classifier, used transcriptomic profiling to classify breast cancers into intrinsic subtypes [3]. Lately, research has progressed beyond broad breast cancer classifications, leveraging advanced multiomics techniques to further refine subtypes within already established clinical and molecular categories. An impressive example is the serial studies carried out in triple-negative breast cancer (TNBC), in which the largest multiomics TNBC database was established so that researchers could systematically characterize the genomic and transcriptomic landscape of TNBC to hunt for potential therapeutic targets [4]. Four distinct molecular subtypes were identified, each with unique therapeutic vulnerabilities. The subsequent FUTURE trial [5] and FUTURE-SUPER trial [6] demonstrated that tailoring therapy based on such molecular subtyping significantly improved clinical outcomes in patients with metastatic TNBC. Recent efforts in molecular subtyping using multiomics approaches have also been applied to other types of breast cancer and followed-up by clinical trials [7]. These multiomics-based advances have substantially improved our understanding of tumor heterogeneity, enabling more precise and personalized treatment strategies to be developed. However, not all identified subtypes necessarily correspond to actionable targets or available therapeutic agents. Moreover, as the incorporation of increasingly complex multimodal datasets becomes more common, the integration strategies and interpretability of multiomics classifications face growing challenges. Nonetheless, continued progress in computational modeling, machine learning and systems biology is steadily improving our ability to extract clinically actionable insights from these data-rich platforms.

Predictive modeling has been used with the aim of developing multi-gene signatures tailored to specific clinical questions, such as determining the necessity of intensified chemotherapy. A landmark in this field was the introduction of the 21-gene assay in 2008, which stratifies patients with estrogen receptor-positive breast cancer based on their risk of recurrence [8]. In recent years, predictive models have advanced rapidly. One example is the homologous recombination deficiency (HRD) score, which is an index based on genome profiling to identify tumors with impaired DNA repair capacity and to indicate potential sensitivity to DNA-damaging agents such as platinum compounds and PARP inhibitors [9]. It integrates three metrics related to the “genomic scar” caused by HRD, namely loss of heterozygosity, telomeric allelic imbalance and large-scale state transitions. Through analysis of large-scale genomic data from tissue samples of ovarian and breast cancers, a threshold was established to define HRD-deficient tumors. Subsequent studies have successfully applied the HRD score in clinical decisions for ovarian cancer. Taking the principle a step further, the phase III PAOLA-1 trial showed that patients could benefit from treatment if their HRD scores were above the threshold, even without *BRCA* mutations, proving its value beyond conventional single gene status [10].

As new technologies come to the fore, we expect continued innovation in this space. As already mentioned, tumor heterogeneity is a key issue in target discovery. Single-cell omics techniques are enabling high-resolution profiling at the cellular level, while spatial omics techniques are adding a complementary dimension by preserving the spatial context of gene expression. Together, they provide a more comprehensive view of tumor heterogeneity, revealing features often lost in bulk methods. Longitudinal liquid biopsy profiling may offer an additional powerful strategy to monitor the dynamic evolution of tumors in real time in a non-invasive way.

As omics data becomes increasingly complex, artificial intelligence (AI) is being utilized to facilitate its analysis, improving both interpretation and clinical decision-making. For example, a recent study used AI-based virtual screening to identify a novel molecule named bemcentinib for the treatment of Parkinson’s disease [11]. This type of “AI for science” research provides a valuable paradigm for cancer research. In particular, given the abundance of large-scale public databases available in the cancer field, AI offers powerful tools to extract actionable targets from these complex and heterogeneous datasets.

Notably, some key challenges remain, especially in terms of the application of these tools. Techniques such as high-throughput sequencing are usually expensive and require advanced equipment and trained personnel, making them less accessible for use in treating patients, especially in less well-resourced regions. These tests also have requirements for sample volumes and processing methods, which can be difficult to satisfy in some cases. In addition, dynamic monitoring of disease is a significant issue, especially for cancer, which requires more non-invasive testing methods. Emerging technologies such as ctDNA analysis, radiogenomics and AI-based predictive tools are helping to lower the threshold for clinical implementation of omics by enabling more accessible, real-time and patient-friendly solutions.

Omics technologies have revolutionized cancer research by enabling target discovery, the refining of molecular subtypes and informing predictive models for clinical decision-making. Despite these successes, challenges such as tumor heterogeneity, data complexity and implementation barriers remain. Integrating multiomics approaches with spatial analysis and AI promises to bridge these gaps, enabling more precise, adaptive and accessible cancer care. Continued innovation will be essential to fully realize the clinical potential of omics-driven precision oncology.

## Abbreviations

AI: artificial intelligence
ctDNA: circulating tumor DNA
HRD: homologous recombination deficiency
TNBC: triple-negative breast cancer
